# Managing River Low Flows to Enhance Instream Vegetation Recruitment

**DOI:** 10.1007/s00267-025-02187-1

**Published:** 2025-05-20

**Authors:** Christopher S. Jones, Scott A. McKendrick, Lyndsey M. Vivian, Piyumi Wijepala, Bryan Mole, Darren White, Joe Greet

**Affiliations:** 1 Department of Environment, Energy and Climate Action, Arthur Rylah Institute for Environmental Research, 123 Brown Street, Heidelberg, VIC 3084 Australia; 2 School of Agriculture, Food and Ecosystem Sciences, The University of Melbourne, Richmond, VIC 3121 Australia; 3 North Central Catchment Management Authority, 628-634 Midland Highway, Huntly, VIC 3551 Australia

**Keywords:** Environmental flows, Germination, Macrophyte, Waterway, Condition

## Abstract

River regulation has reduced natural flow peaks in rivers globally, and in some cases has also reduced the occurrence of low-flows that expose the riverbed. Minimum low-flows are commonly mandated for temperate managed waterways in summer to maintain water quality and aquatic habitat for flora and fauna, at levels which prevent riverbed exposure. Very low flows that allow partial riverbed exposure may have many important roles in naturally impermanent waterways, including promoting plant recruitment. We conducted an in-situ field experiment in a regulated river by drawing down flows for two weeks in austral autumn to facilitate plant recruitment from the riverbed. We also conducted a concurrent ex-situ experiment in controlled conditions using sediment samples from field plots and subjecting them to exposure and inundation treatments. Two-week exposure of riverbed sediments was sufficient to trigger the germination of thousands of flood-tolerant riverine plants in both the in-situ and ex-situ experiments, but aquatic plants showed little response. Terrestrial plant seedlings were uncommon within the river-bed substrate. Seedlings were tolerant of early re-inundation but prolonged inundation resulted in senescence and mortality for non-aquatic plants. Very low flows in rivers for at least two weeks may facilitate recruitment of flood-tolerant riverine plants but the event timing and re-inundation regime will influence the likelihood of successful plant establishment. While there are potential risks for some aquatic taxa by implementing very low flows, this needs to be weighed against the potential benefits of riverbed exposure for promoting important biotic processes including plant recruitment.

## Introduction

Riverine biota are strongly dependent on the flow regime (Bunn and Arthington 2002, Palmer and Ruhi 2019), however the importance of the low-flow component of this regime is often underappreciated (Lake 2003, McMahon and Finlayson 2003). Flow regulation of temperate waterways often results in a flattening or inversion of natural seasonal flow patterns because of both reduced cool-season flow (through reservoir capture) and increased warm-season flow (for human consumptive needs), the latter precluding natural low flow periods (Greet et al. 2013a, Maheshwari et al. 1995). Alternatively, the rapid and frequent variations in flow in rivers regulated for hydropower may also preclude extended periods of low flow (Strom et al. 2012, Bejarano et al. 2020). Environmental flows in temperate rivers are often considered an important tool for ecological restoration via the delivery of elevated cool-season flows (Stella et al. 2010, Tonkin et al. 2020 and Yarnell et al. 2020), but less often are the provision of warm-season low flow periods considered important (McMahon and Finlayson 2003).

Flow regimes drive riverine plant assemblages along an elevation gradient from the channel bed to the floodplain, with wetter conditions closer to the channel favouring non-terrestrial species (Tonkin et al. 2020, Merritt and Cooper 2000). Lateral hydrological gradients influence early plant recruitment stages, likely setting the template for these riparian vegetation patterns (Fraaije et al. 2015, Stromberg et al., 2007). However, flow regulation in rivers has greatly impacted plant recruitment processes in riverine systems (Dixon and Turner 2006, Greet et al. 2011, White et al. 2021). Elevated flow pulses or high-flows in regulated systems are often recommended to stimulate recruitment of riparian plants at higher bank elevations to alleviate the negative impacts of regulation (Merritt et al. 2010, Stella et al. 2010). The provision of low flow periods may be important for instream plant recruitment, richness and cover (Goldenberg-Vilar et al. 2022) but are much less common.

The success of providing high-flows to stimulate recruitment may be limited because different plant functional groups have distinct recruitment strategies, such as seed based or clonal mechanisms. Terrestrial plants typically reproduce via seed, flood-tolerant riverine plants employ seed and/or clonal mechanisms, while aquatic plants commonly reproduce clonally (Barrat-Segretain 1996). Terrestrial plant propagules typically have seasonal germination triggered by rainfall, while flood-tolerant riverine plants often germinate following flood triggers (Naiman et al. 1998). However, in the stream bed, germination triggers for aquatic plants are less-well understood and flow pulses above an already inundated substrate are unlikely to trigger plant recruitment. Instead, reinstating low-flow periods that provide shallower water depths or exposure of sediments in summer may be important for recruitment of aquatic and flood-tolerant plants within or adjacent to the stream bed (Riis 2008; Greet et al. 2013b).

While aquatic plants may thrive in perennial waterways once established, plant colonisation and recruitment is often sporadic and may be inhibited by perennial flows for many instream species (Gurnell et al. 2007b, Riis 2008). The colonisation and establishment of flood-tolerant and aquatic species are determined by the interaction of plant life history processes, flow regimes, substrate conditions, and climate variables. Seasonal flow patterns are likely to be influential for the reproductive output of riverine plants, germination triggers, as well as inundation tolerances (Britton and Brock 1994, Greet et al. 2013b, Main et al. 2022). For example, flooding has a much greater negative impact on plant health in warmer seasons (Vivian et al. 2020) compared to cool seasons (Kitanović et al. 2023). For these reasons, the lack of natural late summer-autumn low-flows in temperate regulated systems may inhibit riverine plant recruitment in the stream bed.

Flow reductions to expose parts of the stream bed late in the warm seasons may be difficult to implement in managed systems because of conflicting management objectives such as the need to maintain consumptive flow volumes, water quality, recreational and social values, and water habitat for fauna and flora (Jones et al. 2023, Tonkin et al. 2021). Minimum low-flows are often implemented to ensure that flows do not go below levels that may put aquatic organisms at risk, such as fish (Göthe et al., 2019, Tonkin et al. 2021). This means that warm-season stream bed exposure in regulated rivers can be rare, with opportunities for sediment exposure to stimulate plant germination lacking. Delivering warm-season flow reductions therefore requires careful planning and management of risks, as well as a shared understanding and coordination between water managers and researchers evaluating outcomes.

We aimed to test the potential value of warm-season low flows to improve vegetation recruitment. Importantly, we considered actions that were feasible within the constraints of a river in which flows are managed for multiple ecological, cultural, social, and financial objectives. We conducted two concurrent experiments: (1) a two-week reduction of flow level within a regulated river of south-eastern Australia; and (2) ex-situ nursery water level treatments of sediment samples from the riverbed of the same locations. For both experiments, we hypothesised that plants with different levels of flood tolerance would germinate from the seedbank in response to sediment exposure but that only aquatic or flood-tolerant plants would survive subsequent re-inundation. Our specific questions were:

1. Can riverbed exposure in summer or early-autumn trigger the germination of flood-tolerant and aquatic plants?
2. Is there a difference in germination and re-inundation response between aquatic, flood-tolerant and terrestrial plants?
3. How can regulated river flows be managed to best support the establishment of new populations of flood-tolerant and aquatic plants?

## Methods

### Study Area

Our study was conducted at eight sites along an 80 km lowland section of the Campaspe River downstream of Lake Eppalock, a 305,000 ML capacity reservoir in south-eastern Australia (Supplementary Fig. S1). The Campaspe River is a tributary of the River Murray within the southern Murray-Darling Basin. The region has a temperate climate with a recent mean annual precipitation of around 500 mm and mean annual temperature of 22 °C (Bureau of Meteorology, Climate Data Online: http://www.bom.gov.au/climate/data/index.shtml).

**Fig. 1:**
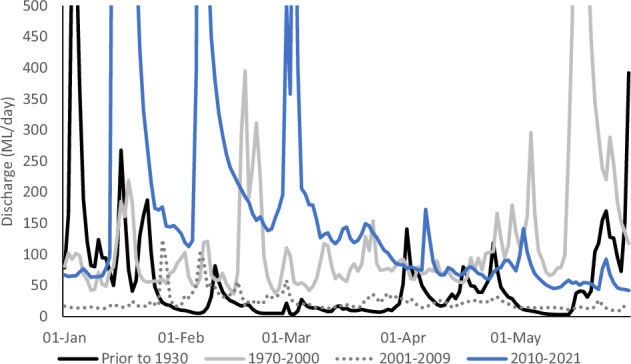
Flow discharge in the Campaspe River in late summer/autumn across four time periods: prior to 1930 indicative of the period prior to large-scale regulation including dam construction, 1970–2000 indicative of the regulated period prior to the Millennium Drought, 2001–2009 spanning the Millennium Drought, and 2010–2021 following the Millennium Drought and the most recent period of environmental water management. Flow measured at Rochester (−36°19'55.0“S, 144°42'03.5”E) and is restricted in the y-axis to highlight variation in lower flow discharges

Prior to river regulation, the Campaspe River had occasionally intermittent flow and would have typically experienced rainfall-driven high-flows in winter and spring, and low-flows (below 10 ML/day) or even cease-to-flows in the late-summer and autumn months (Fig. 1 and see Supplementary Fig. S2 for annual hydrograph). The very low pre-regulation flows were lower on average than during the Millennium Drought (2001–2009) and would have included multiple extended low flow periods (days to weeks) throughout the late-summer and autumn period (Fig. 1). Following regulation, the mean flow discharge rarely drops below 50 ML/day. This lowland section of the Campaspe River occurs within an agricultural landscape (livestock pasture and dryland cropping) with public river frontages maintaining a narrow band of riparian woodland dominated by *Eucalyptus camaldulensis* trees.

### Field Experiment

The field study was centred around an experimental low-flow event delivered in the Campaspe River, which aimed to replicate natural low-flows at the end of the warm season. Naturally variable low-flows, prior to dam construction in 1930, would generally occur from summer to autumn in this system and could result in very low-flows (below 10 ML/day) or cease to flow (Fig. 1).

The experimentally delivered flow event in 2022 was the same across all eight sites apart from a time lag of 2–3 days between the most upstream and downstream sites for the flows to progress further downstream. The experimental low-flow event commenced in early-May 2022 at the end of Austral autumn. This was at the later end of the natural low-flow window, but an earlier flow could not be delivered in that year due to regulation constraints.

**Fig. 2:**
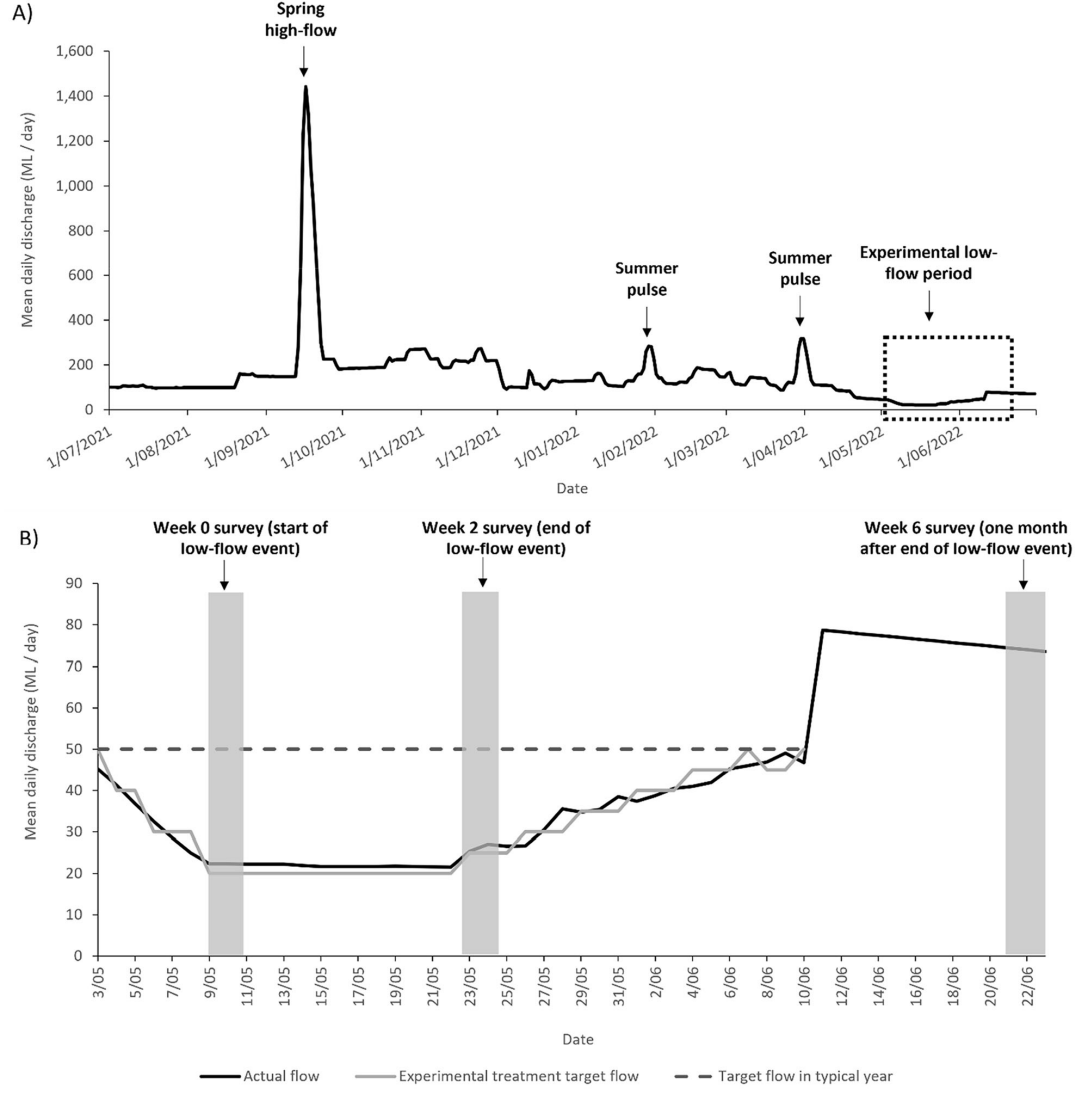
Flow discharge in the Campaspe River measured downstream of Lake Eppalock for (**A**) the year in which the experimental low-flow event occurred (June 2021–July 2022; timing of the experimental low-flow event is indicated by the dotted box) and (**B**) Flow during the experimental low-flow event (3 May–8 June 2022) including the actual flow, the experimental treatment target flow and the target flow in a typical non-experimental year, and the timing of the Week 0, Week 2 and Week 6 surveys

The event involved a gradual one-week flow drawdown from approximately 50 ML/day to a stable low-flow of approximately 15–20 ML/day lasting for two weeks. Flow was then ramped back up to 50 ML/day over two weeks, with a total flow event duration of 36 days (Fig. 2). The two-week period of low-flow was the maximum duration possible given constraints related to potential reduced water quality and fish health concerns. Because of these risks, an operational water quality assessment was undertaken concurrently with the low-flow to identify and manage risks related to aquatic fauna.

Surveys were conducted at the eight sites (Fig. 2) over a 2–3 day window to allow for flow lags. Three surveys were conducted:

- Week 0 (start of low-flow event): 9 May 2022
- Week 2 (end of low-flow event): 24 May 2022
- Week 6 (one month after end of low-flow event): 22 June 2022

Daylength during the survey period ranged from 10 h and 17 min on the 9/05/2022 to 9 h and 33 min on the 23/06/2022 (www.timeanddate.com/sun/australia/melbourne?month=6&year=2022).

**Fig. 3:**
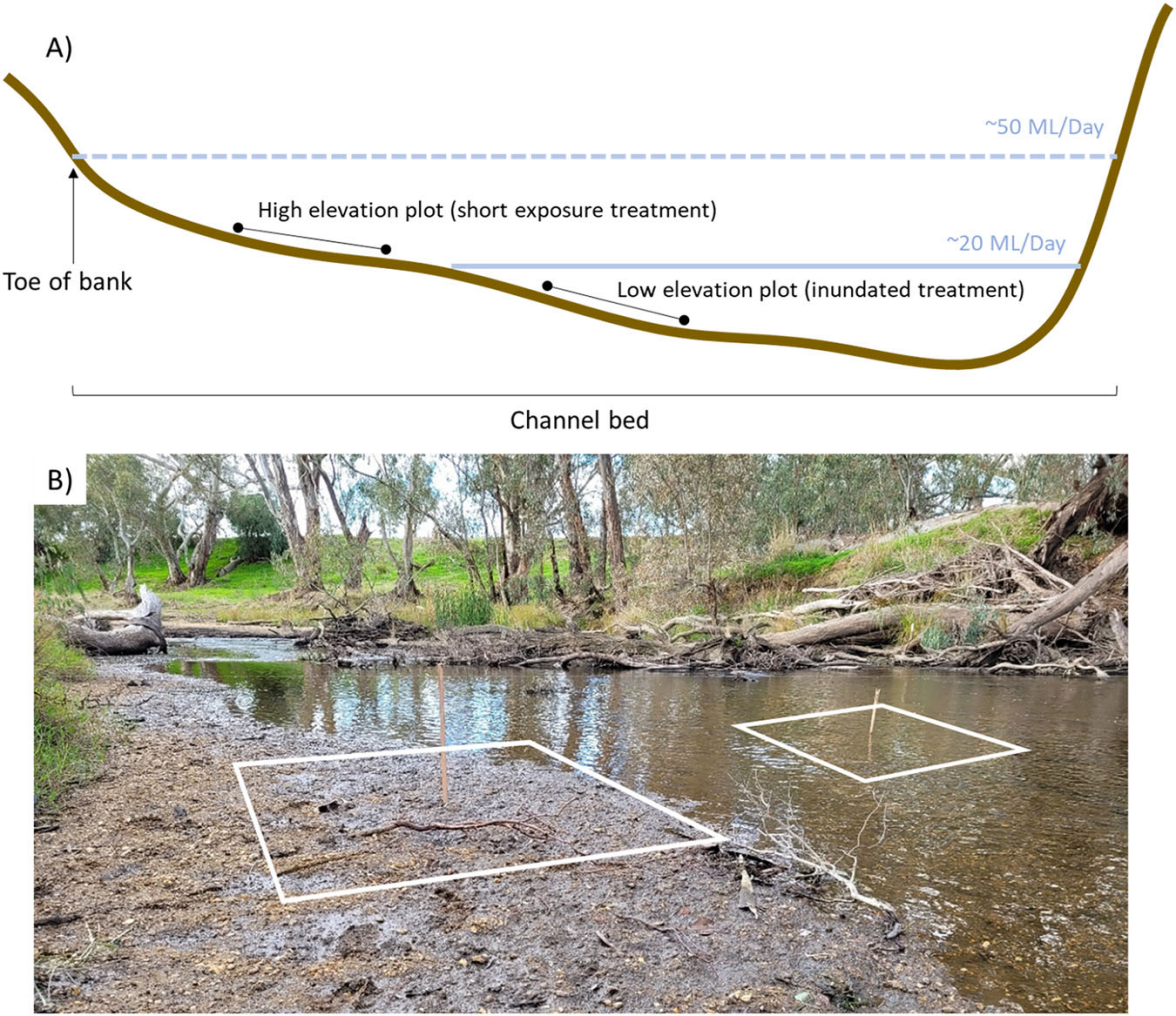
Experimental sampling design for one pair of plots. **A** Schematic of river cross-section showing a pair of plots, with one positioned at a high elevation (short exposure field treatment) and one positioned at a low elevation (inundated field treatment). **B** Example of a plot pair with a wooden stake in the centre of each plot – plot sizes are approximate. Flows were reduced from typical low-flow levels of ~50 ML/day to ~20 ML/day

**Fig. 4:**
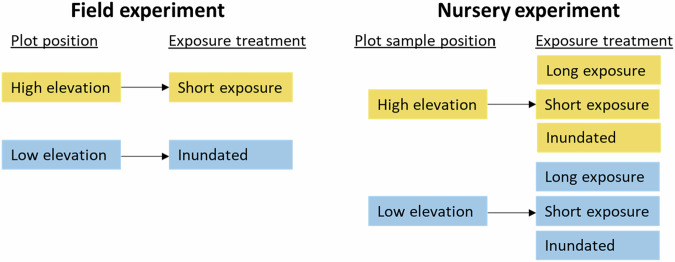
Diagram indicating the treatments applied for the field and nursery experiments

At each of the eight sites, three pairs of 2 m × 2 m plots were established (i.e. six plots per site and 48 plots in total). Paired plots were positioned at two elevations (one high and one low) on the river bank (Fig. 3) to allow the field experiment to compare two treatments (Fig. 4):

- Short exposure: experienced by the high elevation plots which were exposed to air during the two-week low-flow event (hereafter referred to as the short exposure field treatment).
- Inundated: experienced by the low elevation plots which remained shallowly inundated for the duration of the two-week low-flow event (hereafter referred to as the inundated field treatment).

Plot elevations were not measured but were positioned in a standardized way by positioning the high elevation plot just above the water level at the start of the low flow treatment (Fig. 3) while low elevation plots were positioned at a depth that would not be exposed during the treatment. Low elevation plot positions were standardized by water depth at the time of initial surveys and depths ranged from 10–40 cm. At each plot, we counted all seedlings present and identified them to the highest taxonomic resolution possible. Seedlings were typically <1 cm tall and species identification difficult, so plants were often recorded at the genus or lifeform level. Water depth, the cover of vegetation, and dominant substrate type (sand/silt/litter/algae/gravel) were also recorded. Basic soil types were defined by particle size following the Wentworth Scale where clay <0.0039 mm, silt = 0.0039–0.063 mm, sand = 0.06–2 mm, and gravel = 2–64 mm (Bunte 2001). Substrate type was assessed and assigned in the field using simple visual and manual texture inspections, which were sufficient to coarsely categorise substrate type as required for this study.

### Nursery Experiment

An ex-situ nursery experiment was conducted using sediment samples taken from the 48 plots established for the field experiment. Taking the samples from the survey plots was not expected to have any influence on the plot survey results because of the very small volumes of sediment removed, and the benefit of comparing the exact same sediment was considered of greater value. The ex-situ experiment was conducted at the University of Melbourne’s Burnley campus nursery. Sediment samples were collected from the field during the Week 0 survey for the field experiment. Samples were collected using a trowel with depth measurement markings to standardize samples. Samples collected in the water were cupped to retain sediment as it was drawn through the water. At each plot, three approximately 5 cm × 5 cm × 5 cm sediment sub-samples were collected and bulked into a single sample and mixed thoroughly. The bulked samples were kept moist and cool and transported directly to the nursery. The bulked samples from each of the 48 individual plots were then divided equally (by volume) into three treatment samples, resulting in a total of 144 samples.

These samples were subjected to three nursery treatments with the experiment running for 22 weeks (Fig. 4):

1. Long exposure: exposed sediment, kept moist via mist irrigation for the duration of experiment.
2. Short exposure: exposed sediment, kept moist via mist irrigation for two weeks then inundated to a depth of approximately 10 cm for the remainder of the experiment.
3. Inundated: inundated to a depth of approximately 10 cm with no sediment exposure for duration of experiment.

The short exposure and inundated nursery treatments were designed to replicate the short exposure and inundated field treatments (Fig. 4). The inclusion of the long exposure treatment was to test a longer duration of exposure that we were unable to test in the field experiment due to the constraints described above. The samples were assessed for counts of seedlings by species, recorded on five occasions: Week 2, Week 4, Week 8, Week 16, and Week 22.

### Plant Groups

Seedling taxa were categorised into three broad groups based on their flood tolerance (Supplementary Table S2):

- Aquatic (tolerant of permanent inundation and intolerant of dry conditions)
- Flood-tolerant (tolerant of prolonged inundation, including large perennial emergent species)
- Terrestrial (tolerant of brief inundation only, e.g. <1 month)

These definitions and groupings were developed from multiple sources as there is no formalised system of species groupings for river plant species in Victoria. We primarily followed the approach of Pereira et al. (2021), who grouped species recorded in the Campaspe River into two categories (flood-tolerant or terrestrial). However, we added a third group (aquatic), as these species were present in our dataset but were largely absent in the study by Pereira et al. (2021). We also used sources such as the Water Plant Functional Group (WPFG) classification developed by Casanova (2011) for wetland plants in south-eastern Australia and known distributions or affiliations of plants to waterways.

### Statistical Analysis

Field data for extant vegetation cover and substrate type were visually inspected to determine correlations between variables and across sites using linear regression and boxplots. Based on these initial summaries, substrate attributes and extant plant cover were not used in subsequent analyses due to the absence of correlations and no explicit hypotheses relating to these factors.

Data analyses were conducted for the field and nursery experiments to determine the effect of treatments on the total count of seedlings by plant group. All data compilation and analyses were conducted using R version 4.2.0 (R Core Team 2022) through R-Studio (RStudio Team 2020).

Generalised linear mixed models (GLMM) with a Poisson family specification were used to evaluate the effect of treatments on seedling counts for both experiments. A standard value of 1 was added to all datapoints to enable fitting of 0 values, which were common in the inundation treatments only. We ran the model using the *glmer* function in the lme4 package (Bates et al. 2015). Model fits were estimated using the *r.squaredGLMM* function within the MuMIn package (Bartoń 2020) providing marginal and conditional *R*^*2*^ values for each model. Visual inspections of model residual plots and quantile-quantile regressions were done to ensure model assumptions were met.

For the field experiment model, inundation treatment (two levels: inundated, short exposure), plant group (three levels: aquatic, flood-tolerant, terrestrial) and their interaction, were included as fixed factors, as well as survey week. The precise timing of exposure for exposed plots varied between sites by up to two days and some small areas of the exposed plots remained inundated throughout the exposure period, but neither of these factors were considered to have significant or measurable impacts on the analysis. Unfortunately, the Week 6 survey was very difficult to conduct after the flow levels had risen again due to poor visibility, so the data were considered unreliable and were excluded from the analyses. This means that while the raw data are presented for all weeks, the analysis of the response is focused on the first four weeks of data only. Site and replicate plots were included as nested random effects to account for site influences and repeated measures, respectively.

In the nursery experiment model, inundation treatment (three levels: inundated, short exposure, long exposure), plant group (three levels: aquatic, flood-tolerant, terrestrial) and their interaction, were included as fixed factors, as well as survey week and bank position. Site and replicate plots were included as nested random effects.

Post-hoc contrasts between inundation treatments and plant groups were conducted on the GLMMs for both experiments using the *emmeans* function in the emmeans package (Lenth 2020) to fit pairwise contrasts between the interacting fixed factors. Treatment contrasts and their confidence intervals (95%) are provided on the back-transformed scale, providing a multiplicative effect of the treatment contrast. This was done using exponentiated coefficient estimates that indicate the proportional increase in the response for that treatment, after compared to before, which was then centred on zero and converted to a percentage, e.g. 10% increase after treatment. Significance was defined by the threshold of *p* < 0.05.

## Results

### Survey Sites

The eight sites varied in their substrate composition and the initial cover of vegetation (Supplementary Table S1 and Supplementary Fig. S3). In general, upstream sites (Sites 1–3) had more gravel while downstream sites (Sites 4–8) had more sand and silt. Substrate composition varied within sites also, but this variation was generally smaller than between sites. Initial cover of vegetation within quadrats was generally low and variable (mean = 9.2% +/−2.7%) and was dominated by aquatic species such as *Cycnogeton procerum* and *Vallisneria australis* as well as the flood-tolerant species *Phragmites australis*.

### Field Experiment

**Fig. 5:**
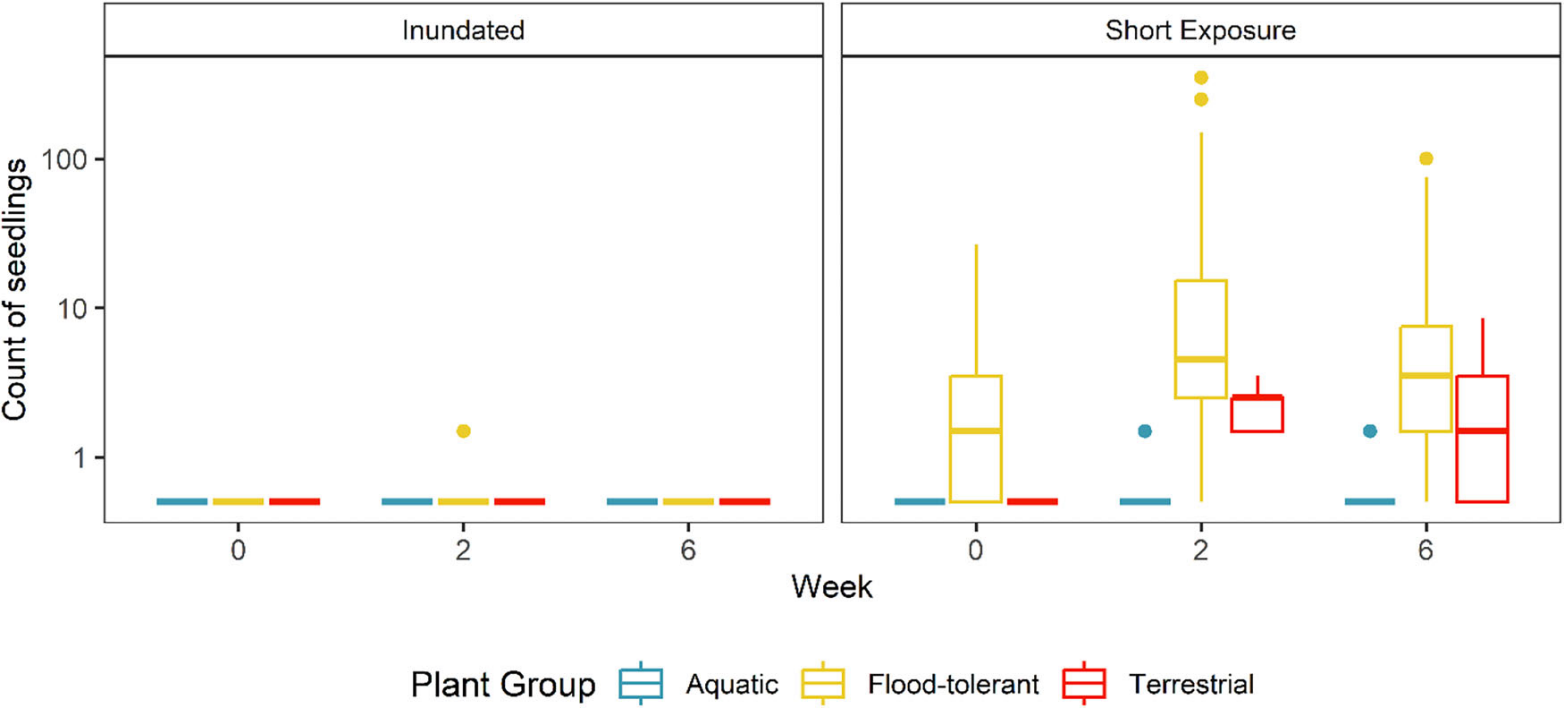
Counts of seedlings across all sites in the field at Week 0 (start of the low-flow period), Week 2 (end of the low-flow period), and Week 6 (one month after end), for the three plant groups (aquatic, flood-tolerant and terrestrial) and the two plot exposure treatments (inundated, short exposure). The y-axis is log scaled to improve data visibility. Boxplots show the median and the range between the first and third quartiles of the data, the whiskers extend up to 1.5× the inter-quartile range

The low-flow event in the Campaspe River that exposed the riverbed substrate to the air resulted in mass germination, particularly of flood-tolerant plants (Fig. 5). In total, during the Week 2 survey, 2082 seedlings were recorded, including 1 aquatic, 2051 flood-tolerant and 30 terrestrial seedlings.

Sediment exposure of two weeks was sufficient to enable the germination and growth of multiple species up to approximately 1 cm tall. Numbers were dominated by flood-tolerant rushes and sedges (*Juncus* spp. and sedge spp., >1400 seedlings across all plots), with the flood-tolerant tree species *E. camaldulensis* also abundant (544 seedlings). Terrestrial grasses and flood-tolerant dicots were present but less common. Only one aquatic plant seedling (*Cycnogeton procerum*) was recorded (during the Week 2 survey). We observed aquatic plant growth of some mature extant plants (rather than seedling recruits) between the Week 0 and 2 surveys, particularly of those that are tolerant of air exposure, e.g. *Cycnogeton procerum* and *Myriophyllum* spp.

The statistical model for the field treatments had a good fit (Marginal R^2^ = 0.79 and Conditional R^2^ = 0.97) and indicated a strong positive effect of exposure on germination. There was a strong positive effect of week on seedling counts, indicating that the Week 2 survey data had significantly greater counts than Week 0 (estimate = 2.10, DF = 261, *p* < 0.001). Treatment contrasts for individual plant groups indicated significant increases for Terrestrial (37%, *p* = 0.019) and flood-tolerant (98%, *p* < 0.001) species but no change for aquatics (2%, *p* = 0.92). There were no obvious patterns in seedling abundance in the field from upstream to downstream, despite variation in substrate composition (Supplementary Table S1, Supplementary Figs. S3 and S4).

Subsequent re-inundation and sediment deposition resulted in difficult survey conditions. Previously exposed plots were partially or fully inundated during the Week 6 survey but most seedlings appeared absent in the inundated areas, potentially drowned, covered in sediment, or washed away. The few remaining and visible seedlings in inundated areas showed minimal growth since the Week 2 survey. Small parts of exposed plots that were still exposed by the Week 6 survey contained numerous seedlings that had grown larger and could be more consistently identified to species level. Visibility in the inundated plots was generally poor during the Week 6 survey due to water depth and turbidity.

### Nursery Experiment

**Fig. 6:**
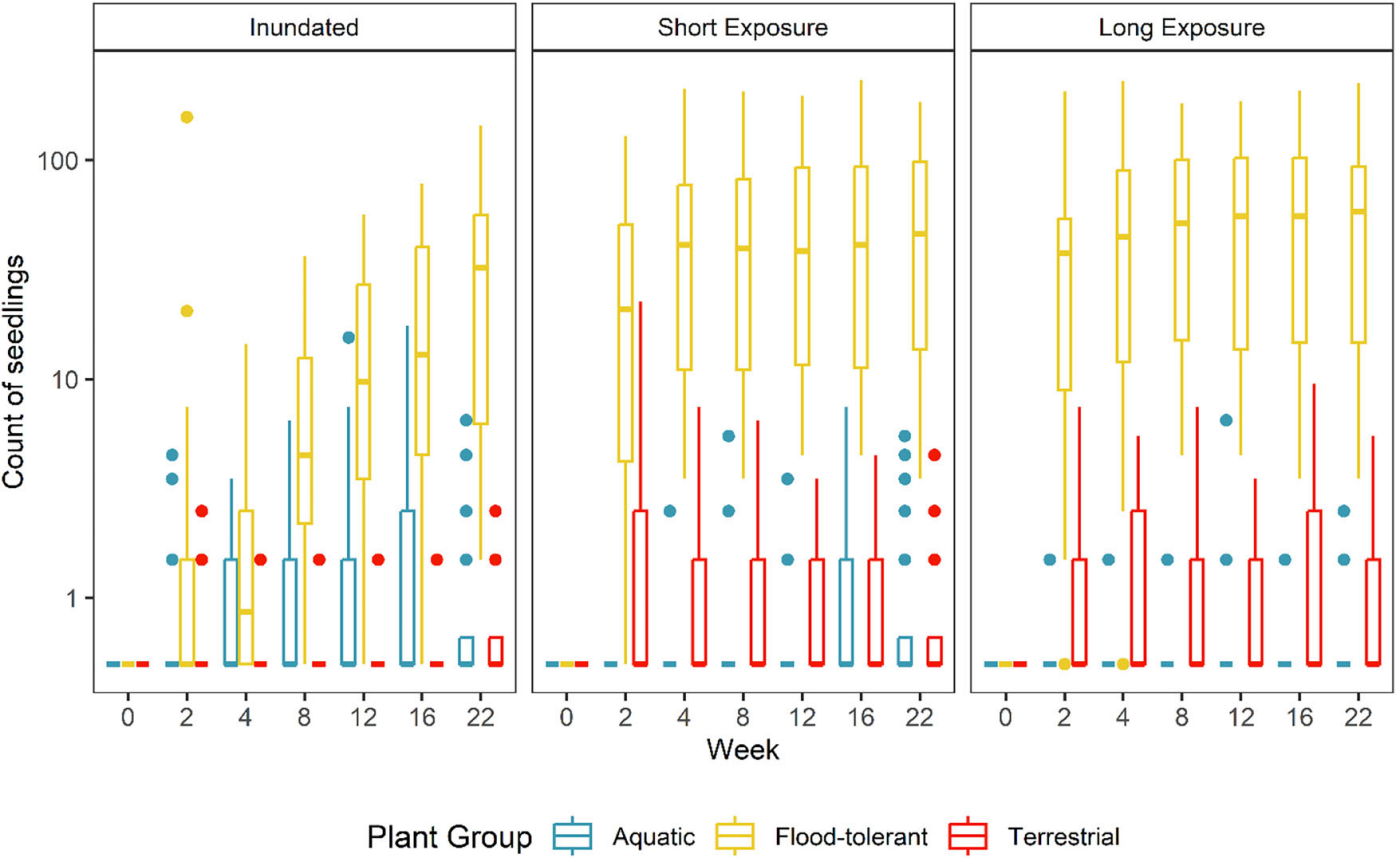
Counts of seedlings for the three plant groups across all nursery treatments by survey week. The y-axis is log scaled to improve data visibility. Boxplots show the median and the range between the first and third quartiles of the data, the whiskers extend up to 1.5× the inter-quartile range

The two low-flow nursery treatments that exposed the riverbed sediment samples to the air (i.e. the long exposure and short exposure nursery treatments) resulted in mass germination of seedlings, particularly of flood-tolerant plants (Fig. 6). In total, 3655 seedlings were recorded during the peak in seedling abundance at Week 16, including 93 aquatic, 3519 flood-tolerant and 43 terrestrial seedlings.

Similar to the field study, sediment exposure of two weeks was sufficient to enable the germination and growth of many species up to approximately 1 cm tall. Seedling abundances were again dominated by flood-tolerant rush (*Juncus* spp.) and sedge (mostly *Cyperus eragrostis*) seedlings after the first two weeks and at the end of the surveys (Table 1). *Cyperus eragrostis* was the most abundant exotic species, while most others were native. In the two exposed treatments, most seedlings appeared within the first two weeks following initial exposure (Fig. 6). Increases in seedling abundance in the inundated treatment was more gradual. Seedlings began to die following prolonged submergence in the inundated and short exposure treatments by Week 22, so the peak in seedling abundance across most species typically occurred at Week 16.

**Table 1:** Total counts of seedlings for the three plant groups and three most abundant taxa across all nursery treatments and sites during peak seedling abundance at Week 16

	High elevation			Low elevation		
	Inundated	Short exposure	Long exposure	Inundated	Short exposure	Long exposure
Terrestrial	1	8	10	1	4	19
Flood-tolerant	280	820	871	230	622	676
Aquatic	46	6	1	23	16	10
*Juncus* spp.	261	559	629	198	470	523
Sedge spp.	14	222	194	25	124	94
*E. camaldulensis*	0	14	15	1	10	13

The flood-tolerant tree, *E. camaldulensis* was again the third most abundant taxon (Table 1) from a total of 36 identified taxa listed in Supplementary Table S2. Terrestrial grasses (e.g. exotic *Bromus* spp. and *Lolium* spp.) and flood-tolerant dicots (e.g. *Persicaria* spp., *Alternanthera denticulata*, and *Rumex* spp.) were present but less common. Emergent flood-tolerant species, such as *Typha* spp. were uncommon, with only 22 seedlings recorded. No aquatic species were recorded in the short exposure or long exposure treatments after two weeks. However, low abundances of four aquatic species were recorded after two weeks, particularly in the inundated treatment: *Myriophyllum* sp., *V. australis*, *Potamogeton ochreatus*, and *Cycnogeton procerum*.

**Fig. 7:**
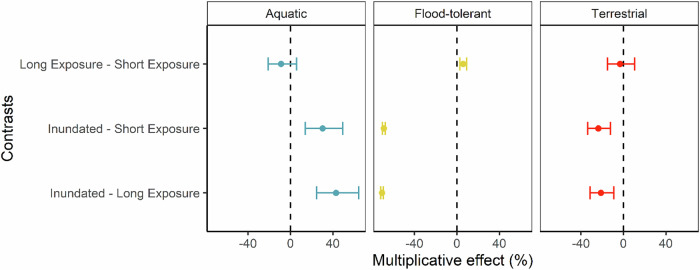
Treatment contrasts from post-hoc analysis of a GLMM for treatment effects on seedling counts within three plant groups. Vertical dashed line at 0% indicates no difference. Error bars indicate 95% confidence intervals

Seedling abundances were higher in the short and long exposure treatments than the inundated treatments for both the combined total of all taxa in the flood-tolerant and terrestrial plant groups, and the three most abundant taxa (*Juncus* spp., sedge spp. and *E. camaldulensis*) (Fig. 7). The seedling ratios for these plant groups in the short and long exposure treatments were similar to the short exposure treatment in the field experiment. In the inundated treatment, seedlings only began to emerge from the Week 2 survey onwards (Fig. 6). These seedlings were predominantly flood-tolerant species, however their abundances were low compared to the short and long exposure treatments. Very few terrestrial species emerged in the inundated treatment (Table 1). Seedlings of aquatic vascular plant species were recorded in the exposure treatments and inundated treatment, but were more abundant in the inundated treatment. It was difficult to determine if the aquatic species arose from seed or vegetative propagules. The charophyte algae *Nitella* sp. was also recorded at relatively high abundance in the inundated treatment and at low abundance in the short and long exposure treatments.

Subsequent re-inundation in the short exposure treatments had little impact on seedling numbers because most seedlings had germinated within the first two weeks of exposure and inundation did not cause rapid mortality of seedlings. Most seedlings remained present and alive but had limited growth for several weeks with mortality then becoming evident in the later parts of the experiment (22 weeks after commencement).

The statistical model for counts of seedlings had a good fit (Marginal R^2^ = 0.73 and Conditional R^2^ = 0.96) and showed that the effect of survey week steadily increased with increasing duration (DF = 3006, *p* < 0.001). Across all treatments and plant groups, the low elevation bank position had 18% fewer seedlings than high elevation plots (*p* < 0.001), but the treatment effects were similar across elevation positions. Treatment contrasts showed that flood-tolerant species seedling counts were significantly more abundant in the short (69%, *p* < 0.001) and long (71%, *p* < 0.001) exposure treatments than the inundated treatment, but long exposure resulted in only 5% more seedlings than short exposure (*p* < 0.001, Fig. 7). Trends for terrestrial species seedling counts were similar but the magnitudes of differences were smaller and there was no significant difference between the short and long exposure treatments (3%, *p* = 0.87). In contrast, aquatic species seedlings counts were significantly less abundant in the short (30%, *p* < 0.001) and long (43%, *p* < 0.001) exposure treatments than in the inundated treatment, and there was no difference between short and long exposure treatments (8%, *p* = 0.42).

## Discussion

Our findings suggest that warm-season low-flows may facilitate the recruitment of flood-tolerant plants, but not necessarily aquatic plants. Our study clearly demonstrated that the exposure of riverbed sediment to air (and consequent increased light and temperature) in both a field and experimental setting resulted in the rapid mass germination of seedlings of flood-tolerant plant species, with most appearing within two weeks of exposure. However, while re-inundation was initially well-tolerated by flood-tolerant plant species, as expected, they showed minimal growth while inundated, and many later senesced. This suggests that low-flows can promote the germination of flood-tolerant plants but that subsequent flow conditions are critical to their recruitment. Aquatic plants did not recruit *en masse* from seed and may require low-flow, but not necessarily exposure of the riverbed, to facilitate recruitment of new plants from either seeds or vegetative fragments (Heidbüchel and Hussner 2019).

The mass germination from seed of flood-tolerant plants in our study is perhaps not surprising given the riverbed is a dynamic store of plant propagules (Gurnell et al. 2007a). The flood-tolerant rushes and sedges that dominated the seedbank are known to produce large numbers of light, buoyant seeds which are relatively long-lived and able to pass in and out of storage within the riverbed and margins through a variety of dispersal processes (Gurnell et al. 2008; Soons et al. 2017). Species such as the flood-tolerant tree species *Eucalyptus camaldulensis* (which was also relatively common) that have relatively short-lived seeds may be seasonally abundant during periods of seed release (Jensen et al. 2008; Greet et al. 2012), requiring low flows that coincide with seed release to promote recruitment.

Seeds of many species of flood-tolerant and terrestrial plants will remain dormant while inundated and germinate following exposure (Gurnell et al. 2007b, Pereira et al. 2021). Some of these dormant seeds will eventually expire if they are not exposed or remobilised via hydrochory (dispersal of propagules via water) to suitable locations for germination (Gurnell et al. 2008). Other seeds will break dormancy even if inundated following other triggers such as temperature and/or light – which we observed in the nursery inundation treatment (Baskin and Baskin 1998, Gurnell et al. 2007b). While access to the air is a significant trigger for germination, winter germination is uncommon for many riparian species as they require temperatures generally greater than 25 °C (Dessent et al. 2019), which might be the cause of the relatively low abundance of dicot seedlings observed within our study. It is likely that the timing of low-flows is important and should not occur too late in the growing season to maximise germination rates of a broad range of species.

We observed few germinants of aquatic or tall perennial emergent aquatic species (e.g. *Typha* spp. or *Phragmites australis*), i.e. the species that are most suited to occurrence on the riverbed. Many aquatic plants in south-eastern Australia, and globally, recruit asexually (via vegetative material) more commonly than sexually (via seed) (Barrat-Segretain 1996). It is possible that recruitment of these long-lived species is sporadic, and results from a combination of disturbance that mobilises plant fragments, and stable or low-flows for subsequent deposition and establishment (Heidbüchel and Hussner 2019, Riis 2008). Recruitment may be a slow process that can be facilitated by flow management or revegetation if colonisation of new populations in the short-term are desired. Nonetheless, we made unquantified observations of growth benefits for aquatic plants from the low-flow event, probably due to increased access to light. This is likely to be particularly beneficial in turbid systems with reduced light availability, which are common in south-eastern Australia (Norris et al. 2001).

Many of the non-aquatic plants seedlings that recruited from the low-flow treatments subsequently senesced. For terrestrial species, this is to be expected and is a good outcome suggesting temporary low-flows will not facilitate colonisation by terrestrial species (Bejarano et al. 2020), but the drowning of flood-tolerant plants is undesirable. Many seedlings can survive several weeks of cool-season submergence (e.g. Kitanović et al. 2023) but are unlikely to survive continuous submergence for >5 months (Garssen et al. 2015), particularly in warmer months (Vivian et al. 2020, Main et al. 2022). Size also matters, with newly established plants that are able to maintain plant parts out of water able to survive much longer (Raulings et al. 2007, Zacks et al. 2018). It is likely that low-flow periods longer than two weeks, or sequential periods of exposure that allow seedlings to grow taller and more resilient to inundation may be required to facilitate establishment of desirable flood-tolerant plants at low bank elevations. In streams across Greater Melbourne, McKendrick et al. (2024) found that more frequent flood events (annually) had a negative relationship with extant flood-tolerant plant diversity and cover, suggesting instream plant communities are indeed shaped by flow components such as low flows. Similarly, in Spain, a comparative study of regulated and unregulated streams indicated that longer periods of low flow increased macrophyte biomass richness (Goldenberg-Vilar et al. 2022).

While comparison of seedling survival from the field and ex-situ experiments is compromised by the difficulties experienced resurveying re-inundated plots in the field, seedling survival following re-inundation was apparently greater in the nursery experiment. This is unsurprising. While prolonged inundation is likely to cause senescence of (non-aquatic) seedlings (and to have done so in both experiments), seedlings in the field were likely lost due to a broader range of processes including the erosive forces of flowing water (Edmaier et al. 2011, Catford and Jansson 2014). For example, in a field experiment along streams subject to hydropeaking (frequent and rapid fluctuations in flow for hydropower), flood-tolerant species managed to germinate and survive early establishment but were eventually killed by the erosive processes triggered by hydropeaking (Bejarano et al. 2020).

### Implications for Flow Management

The absence of low-flows during periods when flows are likely to have been naturally low or even cease-to-flow periods may result in negative outcomes for aquatic and flood-tolerant vegetation. Water managers aiming to improve riparian and instream vegetation communities may need to consider very low-flows that expose parts of the riverbed to promote plant germination and recruitment as a component of a broader flow management strategy to enhance vegetation outcomes. In temperate waterways with artificially stable and elevated summer flow, flow reductions that expose sediments to the air can increase instream plant germination. However, if flow levels return to elevated, stable conditions for extended periods too soon after a germination event, seedling mortality is likely to result. Conversely, repeated or longer periods of very low flow may facilitate plant establishment and mature plant growth, as they provide periods of increased gas exchange and light for rapid growth (Colmer and Voesenek 2009). More research is required to determine the suite of flow conditions and appropriate timing that will promote the establishment of aquatic and flood-tolerant plant species within regulation constraints.

The timing of these trials commencing in late autumn when water temperatures were low and day lengths shorter was less ideal than starting in summer. The timing of the field experiment was constrained by the ability of river managers to deliver low-flows among other competing demands for water delivery in the Campaspe River. Typically, low-flows in the Campaspe River are likely to have occurred naturally in summer and early autumn when day length is longer and water temperature higher. The later timing of our trials may have reduced the germination response of a number of species, particularly aquatics. Repeating these trials in summer and early autumn may provide further insights into how low-flows can promote the recruitment of aquatic vegetation. A valuable comparative study would involve investigations of low flow recruitment processes in similar environments with longer low-flow durations.

Seedlings of plants germinating on the stream bed are vulnerable to drowning (Main et al. 2022), covering by sediment (Lowe et al. 2010), carp disturbance (Roberts et al. 1995), waterbird grazing (Klaassen and Nolet 2007), livestock impacts (Jones and Vesk 2016), and erosion (Kui et al. 2019). Establishment of seedlings to mature individuals that are more resilient to these flow and non-flow disturbances can be difficult and it is likely that flow management would need to co-occur with management of non-flow threats to achieve benefits for riverine vegetation (Stewardson et al. 2017, Tonkin et al. 2020). The application of other management actions such as reducing livestock grazing pressure are also likely to help to maximise seedling establishment and the health of instream plants following mass recruitment events.

It is important to be aware that the provision of periods of very low-flows for plant recruitment benefits would need to be weighed up against any potential risks in meeting other, non-vegetation objectives. Aside from consumptive and recreational needs, elevated low flows in summer are often recommended to maintain water quality, appropriate habitat for aquatic fauna such as fish, or maintaining an open thalweg (North Central CMA 2014, Göthe et al. 2019, Tonkin et al. 2020). Nonetheless, where variable low flow periods (or even cease-to-flow periods) would naturally occur but have been lost from a system through regulation, it is expected that aquatic biota endemic to that system will be resilient to low-flow periods and may benefit from them (e.g. through greater instream vegetation cover) (McMahon and Finlayson 2003). Because of these risks, an operational water quality assessment was undertaken concurrently with the experimental low-flow in the river, which resulted in no detection of risks such as low dissolved oxygen levels. Fortunately, the process of facilitating recruitment (if successful) can result in long term outcomes (years to decades) from a brief period of intervention (<1 season). This means that the outcomes can be great without the need for annual application of the low flow period, and subsequently risks can be mitigated even further by providing the treatment at long intervals, e.g. every 3–5 years (or as suitable for the specific system).

This study was conducted on a single waterway but it has clear and relevant implications for other regulated waterways throughout the region and globally. The application of very low flow treatments as used in this study are likely relevant to any regulated waterways where very low flows can be delivered, are likely to provide a benefit, and are unlikely to pose high risks to other values. Individual waterways would need to be assessed against these criteria to determine if the proposed management action was suitable. Changes to constraints, such as shifting the timing of drawdown to warmer months and preventing subsequent prolonged stable water levels, would enable these flows to be delivered and beneficial in different systems. Revegetation of aquatic plants might be a more effective way to restore instream vegetation in systems where plants are absent and flows are heavily regulated.

## Conclusion

Low flows that expose the riverbed are likely to stimulate mass seed germination of flood-tolerant plant species. However, a single brief exposure event followed by stable elevated flow is unlikely to yield substantial benefits as many seedlings will be drowned or washed away. Modifications of the delivery of the low flow period, such as earlier timing and slower rising flows, may result in successful recruitment of plants. Aquatic plant species are unlikely to recruit *en masse* from these events but very low-flows may provide appropriate conditions to maximise establishment following periods of propagule (both sexual and asexual) dispersal, e.g. after floods. We suggest that environmental flow management requires a greater appreciation of the suite of flow conditions, including low-flows, that promote the establishment and growth of aquatic and flood-tolerant plant species.

## Supplementary information

Jones_Low flow recruitment_Online Resources_revised

## Data Availability

Summary data is provided within the manuscript or supplementary information files, and full data files are available via an OSF online repository: https://osf.io/4ka2t/.
