# Supplementary material for: Managing River Low Flows to Enhance Instream Vegetation Recruitment
Source: Environ Manage. 2025 May 20;75(7):1845–58. doi: 10.1007/s00267-025-02187-1 (PMC12228589; doi:10.1007/s00267-025-02187-1)
Supplement: Supplementary file 1 — Jones_Low flow recruitment_Online Resources_revised [file 267_2025_2187_MOESM1_ESM.docx]

Managing river low flows to enhance instream vegetation recruitment

Christopher S. Jones^1,2*^, Scott A. McKendrick^2^, Lyndsey M. Vivian^1^, Piyumi Wijepala^2^, Bryan Mole^1^, Darren White^3^, Joe Greet^2^

^1^Arthur Rylah Institute for Environmental Research, Department of Environment, Energy and Climate Action, 123 Brown Street, Heidelberg, Victoria 3084

^2^ School of Agriculture, Food and Ecosystem Sciences, The University of Melbourne, Richmond, Victoria 3121

^3^ North Central Catchment Management Authority, 628-634 Midland Highway, Huntly 3551

*Corresponding author.

Online Resources


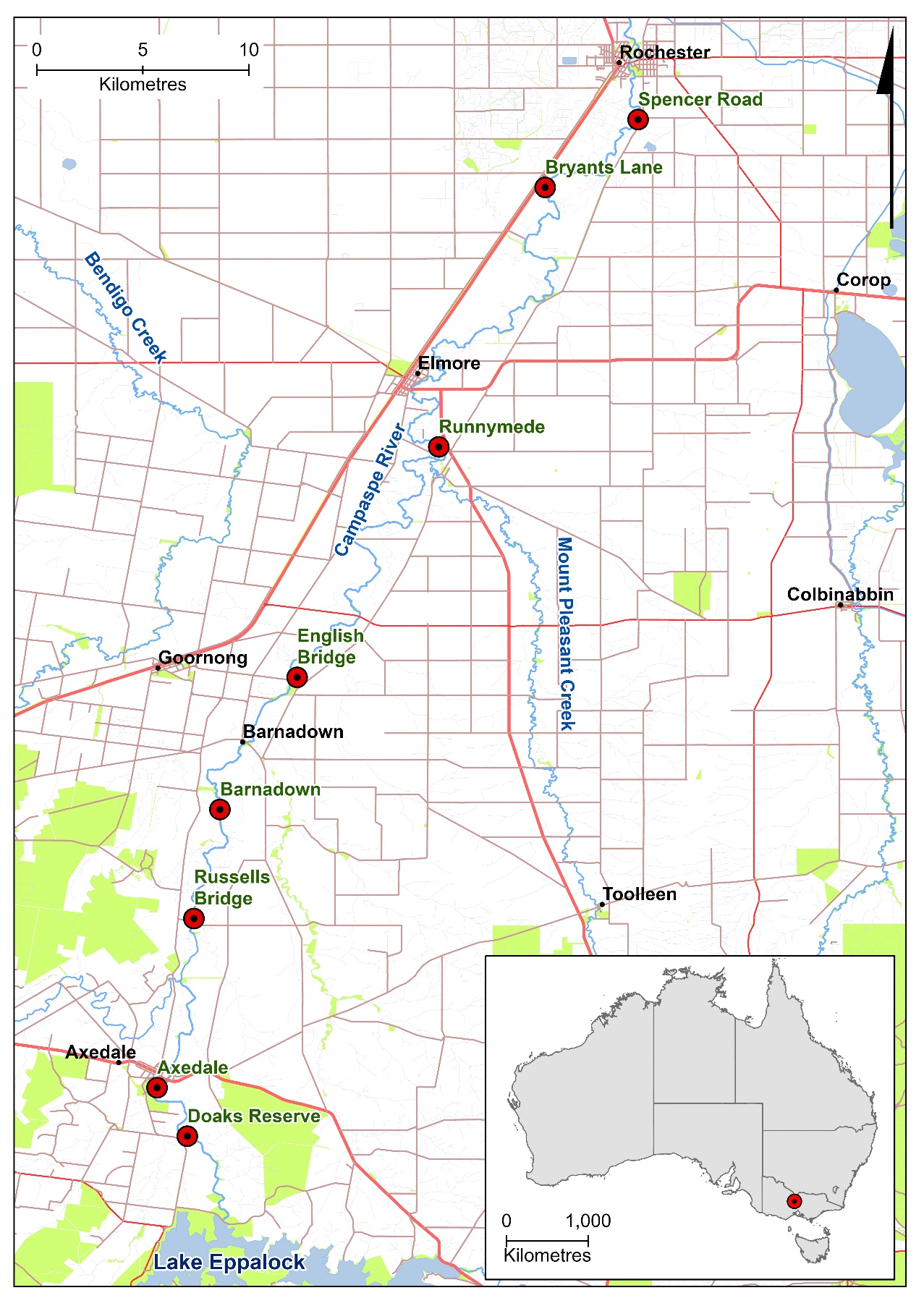


**Fig. S1** Map of the study area indicating the eight sample sites along the Campaspe River downstream of the artificial reservoir, Lake Eppalock.

**Fig. S2** Flow discharge in the Campaspe River across four time periods: prior to 1930 indicative of the period prior to large-scale regulation including dam construction, 1970-2000 indicative of the regulated period prior to the Millennium Drought, 2000-2009 spanning the Millennium Drought, and 2010-2021 following the Millennium Drought and the most recent period of environmental water management. The period of interest for this project is late summer to autumn, when historically flows were low (black line). In the most recent period of environmental water management, flows in late-summer to autumn are elevated compared to historically due to human consumption. Note that the three large flow peaks in the most recent period of environmental water management are due to naturally-occurring floods during early 2011. Flow measured at Rochester (-36°19'55.0"S, 144°42'03.5"E).

**Table S1** Riverbed substrate and vegetation cover averaged within sites.

|  | **Riverbed surface substrate %** | | | | |  |
| --- | --- | --- | --- | --- | --- | --- |
| **Site number** | **Clay** | **Silt** | **Sand** | **Gravel** | **Litter** | **Vegetation cover %** |
| 1 | 0 | 2 | 20 | 75 | 3 | 7 |
| 2 | 0 | 7 | 30 | 60 | 3 | 2 |
| 3 | 0 | 0 | 35 | 60 | 5 | 15 |
| 4 | 0 | 10 | 75 | 5 | 10 | 6 |
| 5 | 0 | 20 | 60 | 15 | 5 | 3 |
| 6 | 0 | 35 | 50 | 0 | 5 | 0 |
| 7 | 0 | 6 | 90 | 1 | 3 | 5 |
| 8 | 2 | 10 | 80 | 1 | 5 | 5 |

*
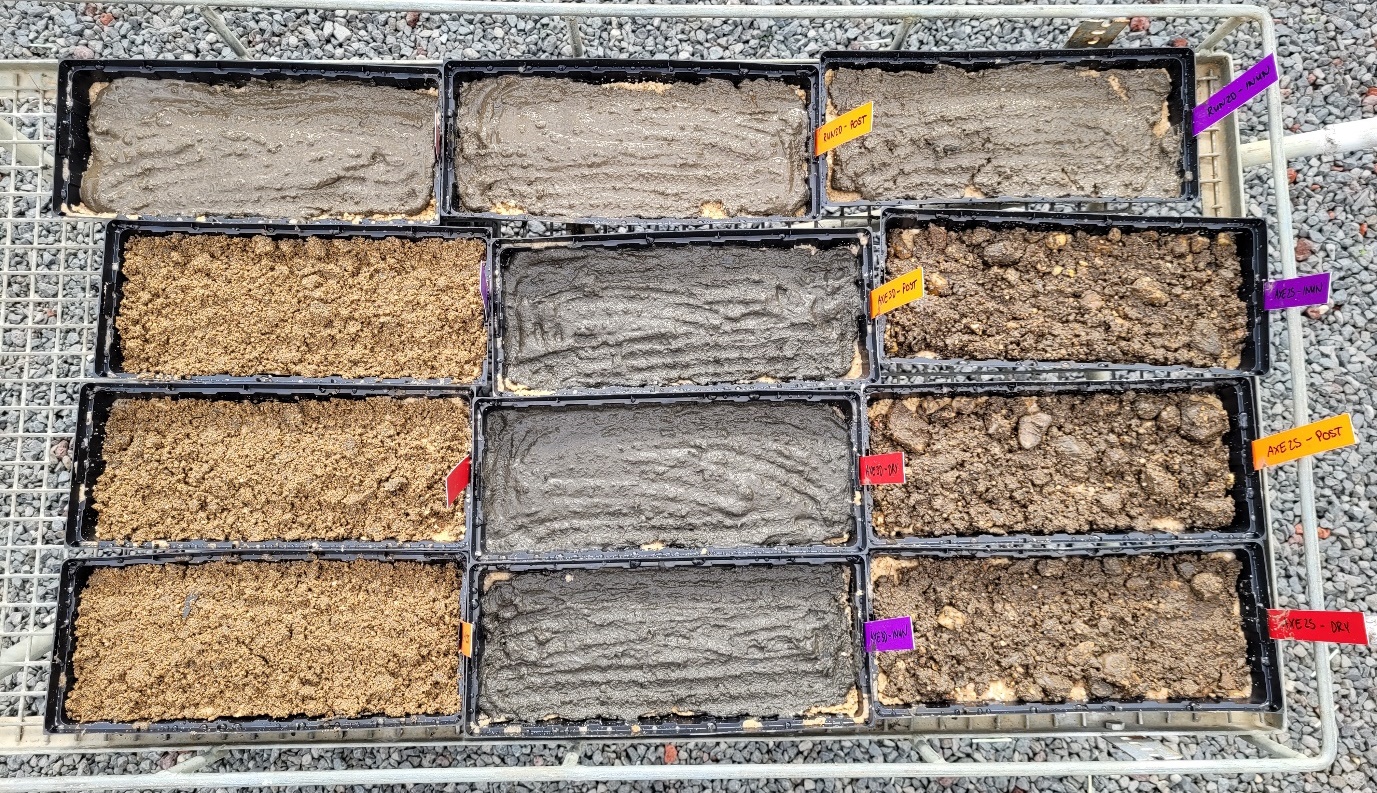
*

**Fig. S3** Photo of sediment samples from different sites locations throughout the study area. Samples show different colours and textures associated with different proportions of sand (left, yellows), silt (centre and top, greys), and gravel (right, browns).


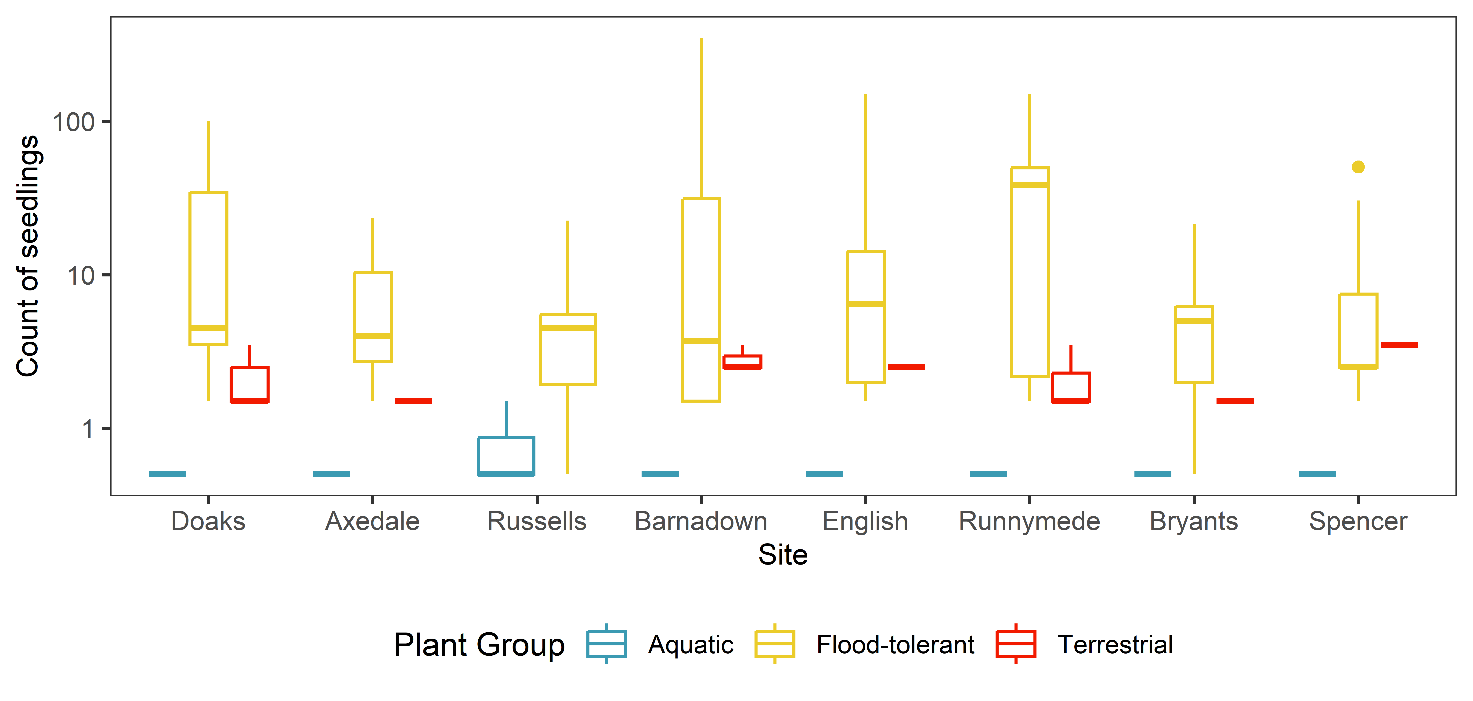


**Fig. S4** Counts of seedlings in the short exposure plots at the end of the low flow period (Week 2) for each field survey site, ranked upstream (left) to downstream (right). The y-axis values are log scaled to improve data visibility. Boxplots show the median and the range between the first and third quartiles of the data, the whiskers extend up to 1.5 x the inter-quartile range.

**Table S2** Summary of all species recorded within the nursery experiment and their maximum seedling count in a single survey week across all samples, sites and treatments.

| **Species** | **Plant Group** | **Maximum count** |
| --- | --- | --- |
| *Alternanthera denticulata* | Flood-tolerant | 18 |
| *Carex fascicularis* | Flood-tolerant | 1 |
| *Characeae* sp. | Aquatic | 76 |
| *Cycnogeton procerum* | Aquatic | 1 |
| *Cyperus eragrostis** | Flood-tolerant | 640 |
| *Dicot* (Unidentified)* | Terrestrial | 65 |
| *Epilobium billardiereanum* | Terrestrial | 5 |
| *Erigeron bonariensis** | Terrestrial | 1 |
| *Eucalyptus camaldulensis* | Terrestrial | 73 |
| *Euchiton japonicus* | Terrestrial | 15 |
| *Glossostigma cleistanthum* | Flood-tolerant | 13 |
| *Grass* (Unidentified)* | Terrestrial | 22 |
| *Isolepis* sp. | Flood-tolerant | 36 |
| *Juncus articulatus** | Flood-tolerant | 79 |
| *Juncus* spp. | Flood-tolerant | 2954 |
| *Lachnagrostis filiformis* | Terrestrial | 32 |
| *Lolium* sp.* | Terrestrial | 7 |
| *Myriophyllum* sp. | Aquatic | 9 |
| *Paspalum dilatatum** | Terrestrial | 3 |
| *Paspalum distichum** | Flood-tolerant | 1 |
| *Periscaria lapathifolia* | Flood-tolerant | 11 |
| *Persicaria decipiens* | Flood-tolerant | 48 |
| *Persicaria hydropiper* | Flood-tolerant | 4 |
| *Persicaria prostrata* | Flood-tolerant | 15 |
| *Potamogeton ochreatus* | Aquatic | 9 |
| *Ranunculus* sp. | Flood-tolerant | 1 |
| *Rumex* sp. | Flood-tolerant | 9 |
| *Schoenoplectus tabernaemontani* | Flood-tolerant | 18 |
| *Solanum nigrum** | Terrestrial | 1 |
| *Symphyotrichum subulatum** | Terrestrial | 8 |
| *Trifolium sp.** | Terrestrial | 2 |
| *Typha sp.* | Flood-tolerant | 19 |
| *Vallisneria australis* | Aquatic | 5 |
| *Verbena officinalis* | Terrestrial | 1 |

** Indicates exotic species*
